# Exploring the Potential of Non-Cellular Orthobiologic Products in Regenerative Therapies for Stifle Joint Diseases in Companion Animals

**DOI:** 10.3390/ani15040589

**Published:** 2025-02-18

**Authors:** Maria Guerra-Gomes, Carla Ferreira-Baptista, Joana Barros, Sofia Alves-Pimenta, Pedro Gomes, Bruno Colaço

**Affiliations:** 1 i3S—Institute for Research and Innovation in Health, Universidade do Porto, Rua Alfredo Allen 208, 4200-135 Porto, Portugal; mguerragomes@gmail.com (M.G.-G.); joana.barros@ineb.up.pt (J.B.); 2 BoneLab, Faculdade de Medicina Dentária, Universidade do Porto, Rua Dr. Manuel Pereira da Silva, 4200-393 Porto, Portugal; csbaptista93@gmail.com (C.F.-B.); pgomes@fmd.up.pt (P.G.); 3 CECAV—Veterinary and Animal Research Centre UTAD, Universidade de Trás-os-Montes e Alto Douro, Quinta de Prados, 5000-801 Vila Real, Portugal; salves@utad.pt; 4 Associate Laboratory for Animal and Veterinary Sciences (AL4AnimalS), Universidade de Trás-os-Montes e Alto Douro, Quinta de Prados, 5000-801 Vila Real, Portugal; 5 LAQV/REQUIMTE, Faculdade de Medicina Dentária, Universidade do Porto, Rua Dr. Manuel Pereira da Silva, 4200-393 Porto, Portugal

**Keywords:** companion animals, stifle joint, cell-free therapy, osteoarthritis, cruciate ligament disease, meniscus, patellar luxation

## Abstract

Stifle joint diseases present a significant challenge in companion animals that often lead to hind limb lameness, with osteoarthritis being a prevalent degenerative condition causing pain and reduced mobility. Regenerative medicine offers a promising avenue for improving treatment outcomes, with a range of emerging therapies showing potential to alleviate symptoms and promote joint health. Among these, hyaluronic acid and platelet-rich plasma have been widely used as intra-articular treatments to enhance joint lubrication, reduce inflammation, and provide symptomatic relief. Interleukin-1 receptor antagonist protein, autologous conditioned serum, and autologous protein solution represent the next generation of regenerative therapies, offering more disease-modifying effects by inhibiting key mediators of joint inflammation. More recently, the MSC-derived secretome has emerged as an innovative, cell-free approach that leverages the diverse bioactive factors secreted by MSCs to support tissue repair and modulate inflammation. This review highlights the evidence base behind these non-cellular orthobiologic treatments for stifle joint disease, aiming to inform veterinary practitioners and owners about available options and their efficacy in supporting conventional treatments.

## Simple Summary

Stifle joint diseases are a significant problem in companion animal medicine, often causing hind limb lameness due to several disorders that invariably lead to osteoarthritis. Therapeutic options aim to restore joint function and manage symptoms and include pharmacological treatments, nutraceuticals, and physical rehabilitation, allied to several surgical techniques. Regenerative cell-based therapies offer a promising avenue for improving treatment outcomes; however, these therapies are laborious and invasive and require a complex regulatory framework, contrasting with the simplicity of cell-free therapies. This review discusses evidence-based non-cellular orthobiologic treatments for stifle joint disease, aimed at informing veterinarians and owners about available options to support conventional treatments.

## 1. Introduction

Stifle joint disease (SJD) is a significant cause of hind limb lameness in companion animals, including dogs, cats, and horses. The stifle joint is a complex structure comprising several key components, such as the cranial cruciate ligament (CCL), menisci, and collateral ligaments, which play vital roles in maintaining joint stability and function [1]. When these structures are compromised, they lead to overall joint dysfunction, reduced mobility, and pain, often progressing into osteoarthritis (OA)—a chronic, degenerative condition characterized by synovitis and breakdown of articular cartilage [2]. This degeneration further impacts the animal’s mobility and quality of life. SJD encompasses a range of conditions, including ligament injuries, meniscal tears, and patellar instability, all of which can lead to chronic inflammation, progressive cartilage degradation, and the eventual onset of OA [3]. These interrelated conditions highlight the need for comprehensive management strategies to address both joint dysfunction and long-term degeneration.

CCL disease is one of the most common causes of hind limb lameness in dogs [4], while it is relatively uncommon in horses and reported much less frequently in cats, with the literature limited to a few cases [5,6]. In canines, the CCL tends to degenerate or weaken over time due to genetic predisposition, conformational abnormalities, and/or immune-mediated processes within the joint [7,8]. Traumatic ruptures are also reported, though less frequently. Conversely, in horses and cats, CCL rupture is commonly related to traumatic events and/or repetitive stress [9,10]. When the CCL is compromised, the stifle joint is subject to successive cycles of cranial translation of the tibia during the weight-bearing phase of the gait cycle while being interspersed with periods of load reduction during the swing phase. This repetitive stress cycle increases the burden on other structures of the stifle and consequently causes pain [11].

The menisci are two semilunar fibrocartilaginous structures within the stifle joint that serve as passive stabilizers of the joint and shock absorbers, protecting the articular cartilage by distributing loads and stabilizing the joint. Damage to the menisci usually occurs when the weight-bearing joint is subjected to a combined flexion–rotation or extension–rotation stress [12,13]. In dogs and cats, meniscal injuries, specifically meniscal tears, are often correlated with CCL rupture [14,15], whereas horses are more likely to develop primary meniscal damage, independent of CCL compromise [16]. The medial meniscus is especially vulnerable, as it is more firmly attached to the tibia, the joint capsule, and the collateral stifle ligament, which makes it less mobile and more vulnerable to injury during joint instability. The lateral meniscus, in comparison, has more mobility, which generally reduces its susceptibility to injury [17]. Additionally, meniscal mineralization, a pathological condition potentially linked with altered joint biomechanics or microtrauma, has been found in cats and may further complicate joint function [18].

Patellar luxation (PL) is another condition frequently contributing to SJD, causing lameness, discomfort, and degenerative joint disease, more frequently in canines and less often in cats. It occurs when the patella shifts out of its normal position within the trochlear groove of the femur, leading to disruption of the quadriceps complex and mechanical instability [19,20]. Studies indicate that PL can increase stress in the CCL, making it more susceptible to degeneration and eventual rupture [21,22]. In horses, a related condition acknowledged as upward fixation of the patella is a condition commonly seen, particularly in certain breeds such as thoroughbreds. This condition causes the patella to lock in an elevated position, typically during weight-bearing activities such as exercise, significantly impairing mobility and potentially leading to long-term dysfunction if left untreated [23].

Osteoarthritis (OA) is a chronic, degenerative disease characterized by the progressive breakdown of articular cartilage. It has a complex and multifactorial etiology, with a strong genetic component and several risk factors, such as breed, age, weight, and sex [24]. OA leads to comprehensive changes in the articular cartilage’s structure and function being one of the most reported musculoskeletal conditions [25]. Unlike other tissues, articular cartilage lacks direct innervation, dedicated blood supply, and lymphatic drainage. It also presents low metabolic activity and limited proliferation of chondrocytes, factors that limit self-healing capabilities and impact the capacity for spontaneous repair [26]. Therefore, once damage occurs, cartilage lesions are unlikely to heal spontaneously and tend to worsen over time, leading to further joint dysfunction and pain.

Identifying the degree of synovitis is crucial in understanding and managing OA, as it is now well recognized that OA typically starts with disease of the synovial membrane itself, also known as synovium, and is driven primarily by macrophages [27]. The synovium plays a crucial role in maintaining cartilage health by producing synovial fluid (SF), which lubricates the joint, nourishes the avascular articular cartilage, and facilitates smooth articulation [28]. It also contains essential nutrients and hyaluronic acid, which contribute to cartilage resilience and shock absorption [28]. However, in the early stages of OA, synovitis—characterized by inflammation of the synovial membrane—can disrupt this balance. Synovitis leads to the overproduction of inflammatory mediators, such as cytokines and matrix-degrading enzymes, which accelerate cartilage degradation, undermine joint stability, and exacerbate inflammation [29]. These pathological changes disrupt the protective environment of the joint, initiating a cascade of cartilage damage. As OA progresses, subchondral bone undergoes remodeling, leading to subchondral sclerosis, a condition where the bone beneath the cartilage becomes denser and less resilient to mechanical stress [30]. This structural change increases the load on the remaining cartilage, contributing to its wear and degeneration. Additionally, periarticular osteophyte formation, or the development of bone spurs at the joint margins, further alters joint mechanics and can restrict movement, contributing to pain and functional impairment [31]. Assessing the severity of synovitis allows veterinarians to intervene more effectively, tailoring treatments to reduce inflammation, slow disease progression, and preserve joint function, ultimately improving the quality of life for affected animals.

As mentioned before, the complexity of OA, with its multifactorial etiology and variable progression, necessitates a comprehensive evaluation that includes physical examination, imaging studies, and joint-specific assessments such as joint palpation for pain and instability or gait analysis [32]. Establishing baseline metrics such as pain scores, range of motion, and weight-bearing distribution enables veterinarians to track disease progression and evaluate the effectiveness of interventions over time [33,34]. Advanced diagnostic tools like radiography, computed tomography (CT), magnetic resonance imaging (MRI), or ultrasonography help confirm the extent of joint damage and identify concurrent issues such as synovitis or ligament injuries [35]. Regularly monitoring these metrics is critical for refining treatment plans, improving patient outcomes, and providing owners with a clear understanding of their pet’s condition and progress, ultimately enhancing the animal’s quality of life.

Despite all the advances in medical and surgical treatments for joint disorders, once OA is established, it tends to progress and worsen over time. Its prevalence has been reported at 20% among dogs over the age of 1 year [34,36], and between 60% and 90% in geriatric cats [37,38]. In horses, joint injuries represent about 60% of equine lameness cases [39]. Beyond the immediate welfare implications for the affected animals, SJD represents a significant global concern for veterinarians, owners, and breeders alike, given the considerable financial burden associated with treatment plans. As a reference, the global companion animal arthritis market size was valued at USD 2.9 billion in 2021 and is projected to reach USD 5.5 billion by 2031, growing at a compound annual growth rate of 7% [40]. In addition to the financial costs, there is also the emotional toll on owners caring for animals with reduced mobility and chronic pain. This emotional strain, often referred to as caregiver burden, can lead to psychological distress and diminished quality of life for the caregivers themselves [41].

Despite its high socioeconomic impact, the available therapeutic options are mainly symptomatic, focusing on alleviating symptoms (such as minimizing pain and reducing stiffness), maintaining functional capacities, and improving the overall quality of life [42]. The first-line pharmacological treatments include oral analgesics, including nonsteroidal anti-inflammatory drugs (NSAIDs). While NSAIDs can decrease inflammation and modulate pain by inhibiting cyclooxygenases (COXs) enzymes, they do not address the underlying disease activity or facilitate cartilage repair. Also, despite being increasingly selective and safe, their prolonged use is potentially associated with adverse gastrointestinal, renal, and cardiovascular effects [42,43,44]. These are especially important in geriatric cases, which represent the majority of patients with OA, many of them presenting comorbidities, with varying degrees of impairment of renal, hepatic, gastrointestinal, and/or cardiovascular function. Nutraceuticals, such as chondroitin/glucosamine sulfate, omega-3 fatty acids, and beta-glucans, are also gaining popularity due to their affordability and potential to combine strategies to reduce inflammation, pain, and slow disease progression [44]. However, the efficacy of these supplements remains under debate, with inconsistent evidence and limited regulatory oversight supporting their use, raising questions about their therapeutic value [45].

Multimodal therapies take a comprehensive approach to managing SJD by combining distinct therapies and symptomatic drug management, nutraceuticals, physical rehabilitation, weight control, and complementary treatments, such as acupuncture, hydrotherapy, and laser therapy. These aim to improve joint function and decelerate the degenerative process by addressing multiple aspects of joint health. However, implementing such a holistic regimen can be costly, time-consuming, and challenging to sustain in daily routines, particularly in geriatric animals or those that have become non-responsive to conventional therapy [45]. Surgical interventions, such as prosthetic joint replacement, joint resurfacing, osteotomy, arthrodesis, and excision arthroplasty, offer options for restoring mobility. However, these procedures may not halt cartilage degeneration or ensure regeneration. Moreover, the selection of patients for surgery must be judicious, and some surgical interventions may not be suitable in specific cases or affordable for all patients [2].

In recent years, tissue engineering techniques have emerged as promising alternatives to overcome the limitations of traditional treatments and utilize novel engineering and biological methods [46]. Due to its complex nature and high-water content, cartilage repair requires a biomaterial matrix that possesses similar viscoelastic properties, which is why hydrogels have been proposed as potential replacement materials or bio scaffolds [47]. Hydrogels are three-dimensional (3D) networks constructed by the cross-linking of hydrophilic polymer chains, through either covalent, ionic, or physical interactions, that allow for the efficient exchange of oxygen and nutrients, provide structural support for cell growth, and act as a substitute for SF by increasing joint lubrication, which consequently prevents pro-inflammatory cytokines from exerting their effects and potentiating OA [48]. This therapy has been used in humans for a decade, and there are some published papers in equine, as well as some research in canine, with the results supporting the application of a polyacrylamide hydrogel in reducing lameness caused by OA [49,50,51,52]. Some hydrogels are already available on the market, specifically polyacrylamide-based hydrogels. Polyacrylamide hydrogels are synthetic, biocompatible, non-toxic, and non-degradable hydrogels that support cellular growth and integration and possess a permanent and stable augmentation effect due to the constant molecular water exchange with their host tissue [53,54].

Polyacrylamide hydrogels are licensed in equine medicine under the brands Arthramid^®^ Vet (Polyacrylamide hydrogel 2.5%), which is also used in canines, and Noltrex-vet™ (Polyacrylamide hydrogel 4%) [55]. Despite being marketed as having different purposes, with ArthramidVet^®^ being characterized as a mitigator of synovitis and Noltrex-vet™ as a cartilage replacement material, both of these materials have the ultimate purpose of alleviating pain associated with these conditions and slowing down, if not preventing, the progression towards OA.

However, hydrogels have certain limitations that reduce their effectiveness as a standalone therapy for treating OA because, while they can provide temporary relief and mechanical support, their ability to address the underlying causes or progression of OA is limited.

To date, the complete restoration of structurally and mechanically functional articular cartilage remains an elusive goal, as pharmacological, multimodal, and surgical approaches have not been able to fully achieve these outcomes. This demonstrates the inherent complexity and challenges of the regenerative cartilage treatment. In response to these limitations, there has been growing interest in the use of orthobiologics—a class of biological therapies that leverage organism-derived substances to enhance and accelerate healing. Orthobiologics include a diverse range of products, such as growth factors, bioactive molecules, extracellular vesicles, and cellular components, which are designed to potentially promote tissue repair, reduce inflammation, and support regeneration [56,57]. Among these, cell-based regenerative therapies have emerged as promising alternatives to the treatment of joint disorders in the veterinary setting [58]. These therapies stand out because they hold the potential to go beyond symptomatic relief by addressing the underlying pathology of the disease. By modulating cellular and molecular processes, they can promote tissue repair and regeneration, potentially offering a more comprehensive and long-term solution for joint disorders. These approaches have been mostly centered on the use of precursor populations, particularly mesenchymal stem/stromal cells (MSCs), which are at the forefront of regenerative medicine due to their well-established potential to differentiate, secrete bioactive factors, and modulate immune responses [59,60]. Mesenchymal stem cells (MSCs) are widely studied for their potential in regenerative therapies, with their ability to differentiate into three lineages—osteogenic, adipogenic, and chondrogenic—serving as a hallmark of their plasticity; in laboratory settings, this trilineage differentiation is often used as a standard test to confirm the multipotent nature of MSCs [61,62]. However, evidence has shown that this differentiation capability is not the primary mechanism of action in MSC-based therapies. Instead, their therapeutic effects are largely attributed to their paracrine activity, which involves the secretion of bioactive molecules such as cytokines, growth factors, and extracellular vesicles that modulate the local microenvironment, promote tissue repair, reduce inflammation, and stimulate the body’s intrinsic healing processes [63]. This shift in understanding highlights that the clinical efficacy of MSCs arises more from their ability to influence their surroundings than from their direct differentiation into specific cell types, marking a significant paradigm shift in regenerative medicine.

To date, in veterinary medicine, MSCs have been isolated from various tissues, such as bone marrow, umbilical cord/umbilical cord blood, placenta, peripheral blood, and adipose tissue [64]. Among these, bone marrow and adipose tissue are the most common tissues isolated for MSCs in veterinary medicine [58].

Cell-based therapies include MSC products, such as bone marrow aspirate concentrate (BMAC), cultured bone marrow-derived stem cells (BMSCs), and cultured adipose tissue-derived stem cells (ADSCs). MSCs appear promising for treatment of OA; however, they are not currently approved by the Food and Drug Administration (FDA) or many other regulatory bodies for clinical use [65]. BMAC is obtained by centrifugation of bone marrow aspirate (BMA) and has been used as a direct joint injection method for early OA repair [65,66]. Compared to BMA, BMAC contains a higher number of progenitor and nucleated cells, as well as cytokines, growth factors, and platelets [67]. However, BMAC has fewer MSCs than those obtained through the expansion of BMSCs and ADSCs in culture [65]. However, cell-based regenerative therapies, while offering promising potential for tissue repair and regeneration, also carry certain drawbacks, especially when compared to cell-free regenerative approaches. One significant issue is the inherent complexity and invasiveness of these therapies, which often requires the isolation, expansion, and processing of cells prior to administration. This can be a time-consuming and costly process, involving invasive procedures for tissue collection, such as bone marrow or adipose tissue harvesting, which carry risks of infection, discomfort, and potential damage to the harvest site, which may limit the therapy’s practicality [68]. In addition, MSCs undergo senescence in 30–40 population doublings [58]. Additionally, there is a risk of immune reactions associated with the use of live cells, especially in cases where allogeneic cells are used [69].

In contrast, cell-free orthobiologics offer a simpler and more convenient alternative. These therapies often involve the direct application of growth factors, bioactive molecules, or other signaling agents that promote tissue repair and regeneration without the need for complex cell processing [70]. As a result, cell-free approaches reduce the risk of immune reactions and can be more easily standardized and quality-controlled, resulting in more consistent and reliable treatment outcomes [70]. This simplicity not only enhances their practicality but also makes them a potentially more scalable and cost-effective option for managing degenerative joint conditions. However, there is significant variability among commercial kits and individual patients in the production and quality of cell-free products, such as differences in kit design, processing protocols, and centrifugation parameters, which can lead to inconsistent concentrations of bioactive molecules, making standardization and efficacy comparisons challenging. Additionally, patient factors such as species, size, and health status contribute to variability in the yield and composition of these products. For small animals like cats and small-breed dogs, certain commercial kits may not be feasible due to the volume of blood required for processing, which can limit their application and necessitate alternative approaches or specialized kits designed for smaller patients.

In the current review, we critically evaluate the potential use and evidence base for non-cellular orthobiologic treatments for SJD in companion animals, specifically dogs, cats, and horses. These approaches include intra-articular administration of hyaluronic acid (HA), PRP, interleukin-1 receptor antagonist protein (IRAP), ACS, APS, and secretome-based therapies. As these regenerative treatments become more accessible and widespread, it is essential for veterinary practitioners to have a thorough understanding of the available treatment options, including their mechanisms of action, efficacy, and safety profiles. By consolidating existing knowledge and highlighting key findings, this review aims to assist practitioners in making informed decisions about the most appropriate and effective non-cellular orthobiologic therapies for managing SJD, ultimately improving treatment outcomes and quality of life for affected animals.

## 2. Non-Cellular Orthobiologic Products

### 2.1. Hyaluronic Acid

Hyaluronic acid (HA) is a naturally occurring and abundant molecule found in SF, playing an important role as a lubricant and shock absorber within the joint [71]. For over three decades, HA has been widely used to treat joint disorders [72]. Viscosupplementation with HA for the treatment of joint diseases aims to improve rheology, as previous studies have shown that there is a decrease in the molecular weight and concentration of HA in arthritic joints [73]. HA has also been shown to decrease inflammation within the joint, further displaying chondroprotective effects, specifically enhancing cartilage formation by increasing the synthesis of glycosaminoglycans (GAGs) and reducing the activity and synthesis of degradative enzymes and pro-inflammatory cytokines [74].

The molecular weight (MW) of HA has a major influence on the rheologic properties in SF, as it dictates the HA’s viscoelasticity and results in decreasing joint friction to prevent further cartilage damage [75]. Intra-articular HA is available in a wide range of MWs, with the molecular weight of native HA having been reported to be ∼4–10 *×* 10^6^ Daltons (Da) in humans and 2–3 × 10^6^ Da in horses [76]. While there is no clear definition of high vs. low MW, products with a MW below 1.5 × 10^6^ Da are frequently considered low MW, while products with a MW above 5 × 10^6^ Da are frequently considered high MW [77]. Different MWs of HA lead to varying anti-oxidative and anti-inflammatory properties. A study in dogs showed that a low MW of 0.84 × 10^6^ Da was more effective in reducing inflammatory changes within the synovium compared with high MW (2.3 × 10^6^ Da). This effect was correlated with the extent of HA penetration into the synovium [78]. In horses, intravenous administration of low-molecular-weight HA has been shown to decrease PGE2 levels within the joint [79].

Intra-articular HA has been widely used in the treatment of human joint diseases, with several clinical studies demonstrating relief of joint pain associated with OA [80,81,82,83,84]. Information regarding the effectiveness of intra-articular HA on degenerative joint disease in companion animals is more limited. The first documented use of HA injections in horses and dogs was in 1971, by Rydell and Balazs, in an attempt to treat post-traumatic joint changes, which proved to be effective, leading to its widespread use in veterinary care [85]. Multiple studies have since detailed the outcomes of this therapy, with some demonstrating a positive effect of intra-articular viscosupplementation with HA, while others reveal neutral results (Table 1). Briefly, a study investigating the effects of single and double intra-articular HA injections in dogs with medial patellar luxation (MPL) following surgery demonstrated improved clinical scores and joint homeostasis. No significant differences were found between the two protocols, indicating that both approaches were equally effective [86]. Another study analyzed the effect of a single intra-articular HA injection on postoperative recovery in dogs undergoing stifle surgery for MPL and CCL rupture (CCLR) repair, showing improved weight-bearing in dogs with MPL, while no significant improvement in lameness scores was obtained in dogs with CCLR, suggesting that a single administration may not be sufficient to mitigate the chronic inflammation under CCLR [71]. Similarly, another study that investigated the recovery of limb function following tibial plateau leveling osteotomy (TPLO) in dogs with CCLR, treated with a single intra-articular HA injection, also revealed no additive benefit of HA on recovery, concluding it to be unnecessary considering the lack of benefit observed up to 6 months postoperatively [87].

In the context of osteoarthritis (OA), several studies have been conducted to evaluate the therapeutic potential of hyaluronic acid (HA). One study induced OA in the stifle joints of dogs by CCL transection. The dogs then received five weekly injections of HA, after which SF was aspirated and analyzed. This study concluded that HA intra-articular injections did not significantly alter the volume of SF, nor did they restore the molecular weight or HA concentration to the levels typically observed in healthy canine joints [88]. However, these results contrast with findings from two other studies, conducted on dogs with spontaneous OA, which reported beneficial outcomes. In one study, dogs with arthritis in a single joint (including the shoulder, elbow, carpus, stifle, or tarsus) were treated with two intra-articular injections of high molecular weight HA at three-week intervals, being compared with a group treated with oral carprofen. The results showed that the HA-treated group showed significantly better outcomes, with 58% of the dogs fully recovering, while only 10% showed no improvement [89]. Another study, which assessed the effectiveness of different numbers of HA injections, demonstrated improvements in clinical symptoms such as pain relief and movement [90].

Only one study was identified that evaluated the therapeutic efficacy of HA treatment in cats. This study examined whether elastoviscous hylan, a derivative of HA, influenced joint nociceptor sensitivity and whether restoring elastoviscosity could reduce nerve responses from nociceptive afferent fibers in arthritic joints [91]. While no studies were found detailing the use of HA viscosupplementation in the stifle joints of horses, HA therapy is widely used in equine medicine for the treatment of arthritis. In a study comparing HA vs. polysulfated glycosaminoglycan administered to the carpal, tarsocrural, metacarpo-, metatarso-, and distal interphalangeal joints of horses, those injected with HA showed reduced lameness scores for up to 5 to 7 weeks [92]. In a more recent study, it was investigated the treatment of naturally occurring OA in the middle carpal joint with HA, with 40% of the horses found to be free from lameness six weeks post-treatment, and 20% achieved a complete resolution of pain during carpal joint flexion [93]. Despite this, not all studies have reported such positive outcomes. In a study involving experimentally induced OA in the middle carpal joint, the administration of HA did not result in significant improvements in the lameness score, carpal flexion, or joint effusion when compared to the control group [94]. Similarly, a randomized, placebo-controlled study investigated different doses of HA for the treatment of experimentally induced OA in the intercarpal joint and found that low dosages had no significant effect on lameness scores and joint function. However, higher dosages showed clinical improvement in lameness and joint function, suggesting that the effectiveness of HA may be dose-dependent [95].

Overall, studies on the use of HA present inconsistent evidence regarding the clinical effectiveness of its intra-articular administration. Reports vary between beneficial and neutral outcomes, with different approaches regarding the optimal concentration and frequency of HA administration. These conflicting findings highlight the need for further research to better define the conditions under which HA viscosupplementation is most effective and to standardize dosing regimens for treating joint diseases in companion animals.

**Table 1:** Characteristic features of clinical and pre-clinical studies evaluating the therapeutic efficacy and safety of stifle intra-articular injection of hyaluronic acid for the treatment of joint diseases in companion animals.

Disease Model	Animal	Therapeutic Approach	Findings	Ref
MPL	Dog	Group 1 (*n* = 12) → 1 intra-articular injection of 0.5–1 mL of sodium hyaluronate (concentration and molecular weight not disclosed) Group 2 (*n* = 10) → 2 intra-articular injections of 0.5–1 mL of sodium hyaluronate (concentration and molecular weight not disclosed) Control group (*n* = 9) → No injection	Higher weight-bearing and lower pain on palpation score. Levels of CS-WF6 were lower in the injected group, and the level of serum HA was significantly higher in the non-injected group. No significant difference was shown between one and two injection protocols.	[86]
CCLR and MPL	Dog	0.5 mL of HA (5–7.3 × 10^5^ Da) at a concentration of 10 mg/mL Group 1 (*n* = 21) → 1 intra-articular injection of HA for MPL Group 2 (*n* = 16) → 1 intra-articular injection of HA for CCLR Control group 1 (*n* = 10) → 1 intra-articular injection of saline for MPL Control group 2 (*n* = 7) → 1 intra-articular injection of saline for CCLR	A single intra-articular HA injection had a positive effect on the weight-bearing of dogs with MPL on post-operative days 1 and 7. The lameness score in dogs with MPL, treated with HA injection, was significantly lower than that of the control. In dogs with CCLR, no significant difference was observed between the HA injection group and the control group in terms of clinical outcomes.	[71]
CCLR	Dog	2 mL of HA (3 × 10^5^ Da) at a concentration of 10 mg/mL Group HA (*n* = 21) → 1 intra-articular injection of HA Group PRP (*n* = 21) → 1 intra-articular injection of PRP Control group (*n* = 20) → no injection	No additive effect on accelerating recovery with the intra-articular injection of HA.	[87]
Induced OA by CCL transection	Dog	Group HA (*n* = 7) → 1 intra-articular injection of 10 mg of HA (1.5 × 10^6^ Da) in 0.67 mL of saline Control group (*n* = 7) → 1 intra-articular injection of 0.67 mL of saline	Intra-articular injection of HA did not alter the volume of SF, nor did it affect the molecular weight or concentration of HA in the stifle joints.	[88]
Spontaneous OA	Dog	1 mL of HA for dogs weighing less than 10 kg and 2 mL for dogs weighing more than 10 kg (concentration and molecular weight not disclosed) Group 1 (*n* = 17) → 1 intra-articular injection of HA Group 2 (*n* = 13) → 3 intra-articular injections of HA	Intra-articular HA improves clinical symptoms in dogs with spontaneous OA, and a single injection produced comparable results to three weekly injections.	[90]
Spontaneous OA	Dog	Group 1 (*n* = 20) → 2 intra-articular injections of 3 to 18 mg of HA (4 × 10^6^ Da) (concentration not disclosed) Group 2 (*n* = 16) → carprofen (NSAID) orally, twice a day	The effect of one to three HA injections on joint inflammation can last between 6 and 12 months, suggesting that periodic intra-articular treatments can have the potential to maintain the function of arthritic joints.	[89]
Induced arthritis	Cat	*n* = 20 (no information regarding distribution) Group HA → 1 intra-articular injection of 0.8% elastoviscous hylan (0.5–3 × 10^6^ Da) in physiological buffer solution Control group → 1 intra-articular injection of 0.8% non-elastoviscous hylan (0.5–3 × 10^6^ Da) in physiological buffer solution	Injection of elastoviscous hylan in the inflamed knee joint significantly reduced the inflammation-evoked ongoing and movement-evoked nerve discharges.	[91]

CCL—cranial cruciate ligament; CCLR—cranial cruciate ligament rupture; HA—hyaluronic acid; MPL—medial patellar luxation; OA—osteoarthritis; PRP—platelet-rich plasma; SF—synovial fluid.

### 2.2. Hemoderivatives

Hemoderivatives have gained increasing attention as beneficial therapeutic options in scenarios where efficient, cost-effective, and safe forms of orthopedic interventions are necessary to restore the normal function and structure of musculoskeletal components. These treatments are particularly appealing when conventional therapies are either insufficient or carry significant risks, prompting healthcare professionals to explore innovative and promising therapeutic alternatives. Using the patients’ own biological materials, hemoderivatives offer a safer approach to tissue healing and therapeutic purposes, often with minimal adverse effects, as they reduce the risk of immune reactions or complications associated with synthetic or foreign substances [96]. Presently, several blood-derived products are available for intra-lesional injection, such as platelet-based products, such as platelet-rich plasma (PRP) or leukocyte-based products, specifically interleukin-1 receptor antagonist protein (IRAP), autologous conditioned serum (ACS), and autologous protein solution (APS) [97].

#### 2.2.1. Platelet Concentrates

The primary function of platelets is to stop hemorrhage after tissue trauma and vascular injury, acting not only through the immediate release of a variety of lipid and protein mediators but also through signal-dependent pre-mRNA splicing and the translation of constitutively expressed mRNA [98]. Platelets are small, anucleated cell fragments that contain a variety of bioactive components, housed within alpha granules, including numerous growth factors and mediators such as transforming growth factor (TGF-β), platelet-derived growth factor (PDGF), basic fibroblast growth factor (bFGF), vascular endothelial growth factor (VEGF), epidermal growth factor (EGF), and insulin-like growth factor (IGF-1) [99,100,101]. Many of these growth factors have demonstrated the ability to enhance and regulate processes like cellular migration, proliferation, angiogenesis, and matrix deposition, both individually and synergistically. Such activities are crucial to promote wound healing in distinct scenarios, with potential functional activity on modulating cartilage homeostasis [100]. Additionally, recent studies have highlighted platelets’ additional roles as important modulators of immune responses, particularly in inflammation [102]. This broader understanding of platelet function stems from their ability to interact with various immune cells and release crucial factors that significantly influence inflammatory processes [103]. This evolving understanding has driven clinical advancements, particularly the shift from employing autologous whole blood to utilizing platelet concentrates (PCs) for enhanced biological modulation [104]. PCs, primarily represented by platelet-rich plasma (PRP) and platelet-rich fibrin (PRF), are autologous therapies that utilize the bioactive components found in plasma and platelets—such as cytokines, chemokines, and growth factors—combined with fibrin-forming proteins to create a natural three-dimensional scaffold [105]. In veterinary medicine, the use of PCs began in the field of sports medicine, primarily for the treatment of ligament and tendon injuries [106]. Over time, their application has expanded to include the regeneration of hard and soft tissues, due to the adhesive properties of the fibrin matrix and the abundance of growth factors stored within the platelets [107].

PRP, the most widely used orthobiologic product, is popular due to its accessibility and simplicity in preparation from both an operational and regulatory perspective. PRP is obtained from an individual’s whole blood that is processed through centrifugation to separate its constituents based on density—namely, the plasma, platelets, leukocytes, and erythrocytes [108]. The resulting PRP is a concentrated suspension of platelets in plasma, enriched with the bioactive molecules found in platelet alpha granules. The minimum concentration of platelets that defines human PRP is approximately two to six times more concentrated than whole blood, i.e., >1 million platelets per µL; however, there are no minimum platelet concentrations defined for companion animals [58,109]. Multiple formulations of PRP have been developed and studied; however, other aspects also need to be taken into account. The impact of the various PRP components other than platelets remains a subject of some controversy in the literature, and so leukocyte-rich PRP (LR-PRP) and leukocyte-poor PRP (LP-PRP) have been the focus of debate over the past few years without a consensus. In general, it is believed that erythrocytes and neutrophils should be reduced as they have an inflammatory effect, while the effect of mononuclear cells remains largely unknown [110,111,112,113,114].

The therapeutic effect of PRP is not due to the platelets themselves, but rather their ability to release bioactive molecules upon activation, such as PDGF, TGF-β, and VEGF, which promote tissue repair and cartilage protection [115]. Cellular therapies rely on direct differentiation and integration, whereas PRP works via paracrine signaling, similar to other cell-free biologics, such as APS and ACS. For this reason, the authors have classified PRP as cell-free therapy.

This therapeutic option has reported beneficial effects across various animal studies (Table 2). Three similar studies assessed the effects of a single intra-articular PRP injection in dogs with stifle joints affected by naturally occurring CCLR and/or OA, demonstrating that a single injection of PRP improved joint kinetics for up to 12 weeks [116,117,118]. Two other studies evaluated the anti-inflammatory effects of a single intra-articular PRP injection in a damaged canine stifle joint, and both showed that PRP effectively reduced TNF-α levels [119,120]. Another study investigated the role of PRP in repairing meniscal white-white zone injuries by promoting the proliferation of canine bone marrow-derived mesenchymal stem cells (BMSCs). The study showed that the expressions of type I and type II collagen in the meniscus were significantly enhanced, while the concentration of osteopontin (OPN) in SF was significantly inhibited in the PRP and the PRP + BMSCs groups, demonstrating that PRP injection may be a promising approach for meniscal repair [121]. Other studies have also been conducted to evaluate the efficacy of PRP therapy and its optimal administration frequency. Two studies, conducted on canine stifles affected by CCLR [122] and/or OA [123], assessed the administration of four intra-articular injections of PRP. The dogs with CCLR demonstrated improved locomotion and reduced lameness, while those with OA showed increased collagen and glycosaminoglycan content, as well as the downregulated expression of inflammatory cytokines such as tumor necrosis factor (TNF)-α, cyclooxygenase (COX)-2, interleukin (IL)-1β, interferon (IFN)-γ, and inducible nitric oxide synthase (iNOS). Another study, which administered five doses of intra-articular PRP to determine their effect on CCL healing, meniscal healing, and progression of OA, yielded similar results, with dogs exhibiting significantly less pain, improved lameness, and better function in the affected hindlimbs, as well as less severe synovitis [124]. The only study conducted on the stifle joint of the horse evaluated how PRP affected joint effusion, lameness, and overall joint function. The authors observed significant improvements, which were maintained for eight months post-treatment [125]. However, to date, no published reports have investigated the therapeutic use of PRP in stifle joint disease in cats.

In contrast to the positive outcomes broadly observed, not all studies have demonstrated beneficial effects with PRP administration. Two studies evaluated the efficacy of a single intra-articular PRP injection in a canine stifle affected by CCLR [87] and OA [126]. The study on CCLR concluded that the additional intra-articular injection of PRP had no additive effect on recovery speed, while the study on OA observed [88] no significant changes in inflammatory markers (interleukin-1 beta (IL-1β), IL-6, IL-10, TNF-α, and prostaglandin (PG) E2) in SF of the PRP treatment group, compared to the control group. Another study tested an alternative application method by incorporating PRP into a collagen hydrogel and showed no significant changes in the disease progression [127].

**Table 2:** Characteristic features of clinical and pre-clinical studies evaluating the therapeutic efficacy and safety of stifle intra-articular injection of platelet concentrates therapies for the treatment of joint diseases in companion animals.

Disease Model	Animals	Therapeutic Approach	Findings	Ref
Stifle degenerative disease secondary to CCLR	Dog	(1013 ± 431 × 10^3^ platelets/μL—3 fold increase) Group 1 (*n* = 12) → 4 intra-articular injections of 2 mL of PRP Control group (*n* = 10) → No injection	The dogs demonstrated improved locomotion, associated with reduced lameness and gait disability remaining effective over a period of 3–6 months.	[122]
Spontaneous CCLR	Dog	(433.5 × 10^3^ platelets/μL and 362 × 10^3^ platelets/μL) Group 1 (*n* = 4) → 1 intra-articular injection of 2 mL of PRP Control group (*n* = 4) → 1 intra-articular injection of 2 mL of saline	PRP reduced inflammation by decreasing TNF-α levels.	[120]
CCLR	Dog	(Plaletet concentration not disclosed) Group 1 (*n* = 21) → 1 intra-articular injection of HA Group 2 (*n* = 21) → 1 intra-articular injection of 2 mL of PRP Control group (*n* = 20) → no injection	No additive effect on recovery speed was demonstrated with the intra-articular injection of PRP.	[87]
CCLR	Dog	(Platelet concentration factor was 6.4 ± 0.82) intra-articular application of PRP-collagen hydrogel (*n* = 29)	A single application of PRP-collagen did not significantly alter the progression of the disease.	[127]
CCLR and MPL	Dog	(Plaletet concentration not disclosed) Group 1 (*n* = 6) → 1 intra-articular injection of 2 mL of PRP for CCLR Control group 1 (*n* = 6) → 2 mg/kg of NSAID orally daily for CCLR Group 2 (*n* = 5) → 1 intra-articular injection of 2 mL of PRP for MPL Control group 2 (*n* = 5) → 2 mg/kg of NSAIDs orally daily for MPL	Groups treated with PRP demonstrated significantly decreased levels of TNF-α, while the NSAID group had increased levels.	[119]
CCL transection and meniscal release	Dog	(Plaletet concentration not disclosed) Group 1 (*n* = 6) → 5 intra-articular injections of 2 mL of PRP Control group (*n* = 6) → 5 intra-articular injections of 2 mL of saline	PRP group had significantly less pain, significantly improved lameness and significantly higher function in affected hindlimbs. PRP-treated stifles also showed evidence of CCL repair and less severe synovitis.	[124]
Meniscal white-white zone injury	Dog	(1368.67 ±52.51 × 10^9^ platelets/L) Group 1 (*n* = 6) → 1 intra-articular injection of 2 mL of PRP Group 2 (*n* = 6) → 1 intra-articular injection of 2 mL of MSCs Group 3 (*n* = 6) → 1 intra-articular injection of 2 mL of PRP plus MSCs Control group (*n* = 6) → 1 intra-articular injection of 2 mL of saline	The application of PRP alone or in combination with MSCs promoted the clinical healing of meniscal white-white zone injury, increased the proliferation of MSCs, upregulated collagens’ expressions, and downregulated osteopontin in SF.	[121]
OA due to ruptured, non-stabilized CCL	Dog	(Plaletet concentration not disclosed) Group 1 → 1 intra-articular injection of 2.5 mL of PRP Control group → No injection	A single injection of PRP improved kinetics for a minimum of 4 weeks with some data suggesting an effect of up to 12 weeks.	[116]
Spontaneous OA	Dog	(739,000 ± 365,000 platelets/µL—3 fold increase) Group 1 (*n* = 10) → 1 intra-articular injection of platelets Control group (*n* = 9) → 1 intra-articular injection of saline	Platelet-injected dogs had significantly improved lameness scores, pain scores, and peak vertical force after 12 weeks, compared with pretreatment values.	[117]
Surgically induced OA	Dog	(Plaletet concentration not disclosed) Group 1 (*n* = 6) → 4 intra-articular injections of 1 mL of PRP Group 2 (*n* = 6) → 4 intra-articular injections of 1.0 × 10^7^ MSCs in 1 mL of PBS Group 3 (*n* = 6) → 4 intra-articular injections of 1.0 × 10^7^ MSCs in 1 mL of PRP Control group (*n* = 6) → 4 intra-articular injections of 1 mL of PBS	The lameness score was significantly decreased at 2 months after treatment in the PRP group, with the study suggesting PRP might have a beneficial effect on OA via the stimulation of ECM synthesis, chondrocyte proliferation, and inhibition of inflammatory reaction.	[123]
Spontaneous OA	Dog	(1.42 × 10^6^/µL concentration of platelets—3 to 4-fold increase) Group 1 (*n* = 14) → 1 intra-articular injection of 3–5 mL of PRP Control group (*n* = 6) → 1 intra-articular injection of 3–5 mL of saline	No significant changes were observed in IL-1β, IL-6, IL-10, TNF-α, and PG-E2 levels in SF from the PRP-treated group, although clinical improvement was observed.	[126]
Surgically created osteochondral defect	Dog	(2 × 10^6^/µL concentration of platelets—5.3 fold increase) Group 1 (*n* = 4) → 1 intra-articular injection of 1.5 mL of PRP and a contralateral intra-articular injection of 1.5 mL of saline Group 2 (*n* = 4) → 1 intra-articular injection of 1.5 mL of SVF and a contralateral intra-articular injection of 1.5 mL of saline Group 3 (*n* = 4) → 1 intra-articular injection of 1.5 mL (2 mg/mL) of SVF plus PLGA scaffolding and a contralateral intra-articular injection of 1.5 mL of saline	PRP treatment resulted in improvements in the lameness scores and objective kinetic assessments of function.	[118]
Osteochondritis Dissecans and OA	Horse (*n* = 7)	(Plaletet concentration not disclosed) Group 1 → 4 intra-articular injections of autologous PC Control group → None	There were significant improvements in lameness scores and joint effusion, leading to an eight-month-long sustained improvement in joint function.	[125]

CCL—cranial cruciate ligament; CCLR—cranial cruciate ligament rupture; ECM—extracellular matrix; HA—hyaluronic acid; IL-1β—interleukin-1β; IL-6—interleukin-6; IL-10—interleukin-10; MPL—medial patellar luxation; MSCs—mesenchymal stem/stromal cells; NSAIDs—non-steroidal anti-inflammatory drugs; OA—osteoarthritis; PBS—phosphate buffered saline; PC—platelet concentrate; PG-E2—prostaglandin E2; PLGA—poly(lactic-co-glycolic) acid; PRP—platelet-rich plasma; SF—synovial fluid; SVF—stromal vascular fraction.

An explanation for these variable results could be the lack of standardization in study protocols regarding the preparation methods for PCs. This includes the concentration of platelets, selective blood filtration of leukocytes to obtain leukocyte-poor and erythrocyte-reduced PRP, and activation methods. Additionally, the number and frequency of injections vary, ranging from a single injection to multiple injections over weeks to months.

Despite these constraints, the results from the included clinical studies demonstrated this therapy’s ability to decrease pain and enhance function in stifle joint disease.

Currently, there are some kits commercially available for processing PRP, such as Restigen PRP^®^, among others, that include provisions for obtaining leukocyte-rich or leukocyte-poor PRP. There are reports that document the results with various kits in both canine and equine species, with high variability in platelet, leukocyte, and erythrocyte concentration being common across species and kits [128,129,130].

#### 2.2.2. Non-Platelet Hemoderivatives—Interleukin-1 Receptor Antagonist Protein (IRAP or IL-1-Ra), Autologous Conditioned Serum (ACS), and Autologous Protein Solution (APS)

Biological therapies derived from autologous blood—IRAP, ACS, and APS—have shown promising potential to modulate inflammation in stifle joint diseases (Table 3) [131]. Unlike platelet concentrates that primarily rely on growth factors for the modulation of tissue repair, these non-platelet hemoderivatives take an alternative approach by targeting cytokine and protein pathways to directly counteract the inflammatory processes central to joint degradation. While biomechanical damage is often considered a primary initiator of the pathogenesis of stifle joint diseases, it is the subsequent biochemical signals that amplify inflammation and tissue breakdown, making these pathways potential therapeutic targets. Innovative agents such as proteinase inhibitors, cytokine antagonists, cytokine receptor-blocking antibodies, and growth/differentiation factors are explored for their potential to disrupt these pathways and provide therapeutic benefits [132].

IRAP, also known as IL-1-Ra, is an anti-inflammatory approach that works by counteracting the pro-inflammatory cascade triggered by the cytokine IL-1 [133,134]. IL-1 is a pro-inflammatory cytokine that is abundantly synthesized in the inflamed synovium and appears to be a key driver of protease synthesis, cartilage degradation, and joint tissue catabolism [135]. The release of IL-1, along with other pro-inflammatory cytokines, triggers a cycle of chronic inflammation and joint deterioration, leading to cartilage damage and disease progression in conditions like OA. The IL-1 family of cytokines includes eleven members, but IL-1a, IL-1b, IL-18, and IL-Ra have been thoroughly described and implicated in joint pathological processes [136,137]. Their biological action on OA is enhanced by at least two principal factors: an increased number of IL-1 receptors (IL-1R) on joint cells and a relative deficit in the competitive inhibitor interleukin-1 receptor antagonist (IL-1Ra) [138]. In addition to the IL-1 receptor, IL-1Ra inhibits several other processes involved in the pathogenesis of OA, such as the synthesis of PGE2 in synoviocytes, the production of collagenases by chondrocytes, and the degradation of the cartilage matrix [139]. In horses, IRAP has demonstrated effectiveness, slowing the progression of OA [134,140]. Although there is no proven in vivo study, Wilson et al., in an electronic questionnaire to various veterinary groups practicing equine sports medicine and rehabilitation, showed that IRAP was administered by more than 80% of veterinarians [140]. A study in dogs showed that treatment with IRAP in knee OA reduced both the incidence and size of femoral condyle osteophytes while also decreasing the progression of cartilage lesions. In addition, IRAP treatment led to a significant reduction of collagenase-1 expression in OA cartilage, which demonstrates that IRAP injection can protect against the development of OA lesions [141]. To date, no study has evaluated the efficiency of IRAP administration in cats.

ACS and APS build upon the anti-inflammatory principles of IRAP, expanding therapeutic options to provide potentially more robust, disease-modifying outcomes [142,143]. Both products are obtained from the patient’s blood and administered directly into the affected joint(s).

ACS is produced by the incubation of whole blood with medical-grade glass beads coated with chromium (II) sulfate (CrSO_4_), which activates leukocytes and other cellular elements, resulting in serum enrichment with anti-inflammatory mediators like IL-1Ra, IL-4, IL-10, and IL-13, as well as growth factors such as TGF-β, VEGF, and bFGF [144,145,146,147]. ACS preparation techniques used for application in veterinary medicine include IRAP I™ and IRAP II™ [58]. Several studies have found that an intra-articular ACS injection increases the concentration of IL-1Ra, helping to mitigate joint inflammation [148,149,150]. In a comparative study of two protocols using ACS for intra-articular treatment of equine osteoarthritis, it was found that the success of administering intra-articular ACS injections varies depending on the time interval in which it is administered. The horses in group 1 received three intra-articular ACS injections at weekly intervals, while in group 2 they received the three injections at 2-day intervals. The study showed that group 2 presented a significant decrease in the levels of IL-1ra, IL-1β, C12C, CS 846, and CP II, which indicates a reduction in cartilage degradation processes. Thus, the administration of intra-articular injections of ACS at 2-day intervals seems to be more effective, as it leads to a greater reduction in joint inflammation and cartilage degradation processes [148].

Over a decade after the initial investigation of ACS, a variant of ACS called APS was developed to further enhance the anti-inflammatory and anabolic properties of hemoderivatives for clinical use [131]. APS preparation combines the beneficial effects of ACS, which contains increased levels of IL-1RA and IL-10, with the beneficial effects of PRP, which release a multitude of anti-inflammatory cytokines and growth factors [131]. A commercially available APS preparation (Pro-Stride™ (Mansfield, MA, USA)) has been gaining clinical popularity because the product does not require an incubation period and has been investigated using a single intra-articular injection in horses and dogs [58].

For the preparation of this product, blood is first collected and mixed with citrate dextrose solution (ACD) as an anticoagulant. It is then transferred to a separation device and centrifuged. Then, the intermediate cell suspension (containing platelets and white blood cells) is transferred into an APS concentrator, where it is mixed with polyacrylamide beads and centrifuged again to produce the final, highly concentrated APS product, ready for use [151]. To date, the effect of intra-articular injection of APS has been evaluated in dogs and horses [149,152,153]. Studies have shown that intra-articular injection of APS is effective for the management of OA in dogs and horses, as it reduces lameness scores and improves weight-bearing associated with the affected joints [152,153]. Wanstrath et al. showed that treatment with APS reduced pain and lameness scores and increased force plate values (peak vertical force and vertical impulse) over 12 weeks, compared to the control group (intra-articular injection of saline) [152]. The study in horses also showed that treatment with APS improved the degree of lameness, asymmetry indices of vertical peak force, and range of joint motion in 14 days compared to the control group (intra-articular injection of saline) [153].

In a comparative analysis of ACS and APS, APS contained higher concentrations of anti-inflammatory cytokines (IL-1Ra, sIL-1RII, sTNF-RI, and sTNF-RII) and anabolic growth factors (PDGF-AB, PDGF-BB, TGF-β, and EGF), as well as lower levels of inflammatory cytokines (IL-1β and TNFα) compared to ACS [149,154]. These differences in cytokine and growth factor profiles suggest that APS may offer a more robust anti-inflammatory and regenerative effect.

**Table 3:** Characteristic features of clinical and pre-clinical studies evaluating the therapeutic efficacy and safety of stifle intra-articular injection of interleukin receptor antagonist protein, autologous conditioned serum, and autologous protein serum for the treatment of joint diseases in companion animals.

Disease Model	Animals	Mode of Application	Findings	**Ref**
Surgically-induced OA	Dog	Group 1 (*n* = 6) → 8 intra-articular injections of 2 mg of IRAP Group 2 (*n* = 5) → 8 intra-articular injections of 4 mg of IRAP Control group (*n* = 5) → 8 intra-articular injections of saline	IRAP exerted a dose-dependent protective effect on the development of osteophytes and cartilage lesions.	[141]
Spontaneous OA	Horse	Group 1 (*n* = 6) → 3 intra-articular injections of ACS at weekly intervals Group 2 (*n* = 6) → 3 intra-articular injections of ACS at two-day intervals	Intra-articular injection of ACS lead to a reduction in joint inflammation and cartilage degrading processes.	[148]
Spontaneous OA	Dog	Group 1 (*n* = 10) → 1 intra-articular injection of APS Control group (*n* = 10) → 1 intra-articular injection of saline	APS reduced pain and lameness scores and increased weight-bearing associated with the OA-affected joint at 12 weeks.	[152]
Spontaneous OA	Horse	Group 1 (*n* = 20) → 1 intra-articular injection of APS Control group (*n* = 20) → 1 intra-articular injection of saline	The APS group had significant improvements in lameness score, asymmetry indices of vertical peak force, and range of joint motion by 14 days compared with baseline or control group values.	[153]
Spontaneous articular lameness	Horse	Group 1 (*n* = 6) → 3 intra-articular injections of ACS at approximately 2-week intervals	Higher levels of IL-1Ra and IGF-1	[150]

ACS—autologous conditioned serum; APS—autologous protein serum; IGF-1—insulin-like growth factor 1; IL-Ra—interleukin-1 receptor antagonist; IRAP—interleukin-1 receptor antagonist protein; OA—osteoarthritis.

### 2.3. Secretome

The secretome refers to the collection of bioactive molecules secreted by cells into the extracellular environment, encompassing a wide array of proteins, cytokines, growth factors, and extracellular vesicles (EVs). These factors play critical roles in cellular communication, influencing processes such as tissue homeostasis, immune modulation, and inflammation control, as well as tissue healing and regeneration [155]. Recently, the MSC-derived secretome has emerged as a particularly promising cell-free therapeutic approach in regenerative medicine. By harnessing the potent combination of bioactive molecules naturally secreted by MSCs, this therapy potentially offers targeted regenerative and anti-inflammatory effects without the associated logistical, regulatory, and safety complexities typically associated with traditional cell-based treatments [2]. This has the potential to enhance the management of various degenerative and inflammatory conditions, particularly those affecting the musculoskeletal system.

The potential of these secreted factors to exert paracrine effects has garnered significant interest in veterinary medicine, where several studies support the hypothesis that the bioactive molecules secreted by MSCs alone may be sufficient to heal or prevent damage to the injured tissue [156,157,158,159,160]. While in vitro studies have demonstrated the efficacy of the MSC secretome in promoting tissue healing in veterinary applications [161,162], so far, in vivo research remains limited. To date, only one study (Table 4) has explored the potential of the conditioned medium from MSCs’ cultures (CM-MSCs) for treating joint diseases in companion animals [161]. In this study, dogs affected by naturally occurring OA in their stifle joints were treated with two intra-articular injections of lyosecretome (freeze-dried cAT-MSC secretome) in the right joint and placebo in the left joint, with an interval of 40 days between injections. The results showed that intra-articular injection of CM-MSCs is safe and does not induce systemic adverse reactions, since no significant results were observed in terms of lameness and pain worsening. In addition, no systemic adverse reactions were observed [161]. However, the number of animals was limited, so more studies will be needed to prove the safety and efficacy of lyosecretome in the treatment of OA in dogs. Furthermore, it is important to underscore that treatments involving secretome, EVs, or exosomes have not yet been approved by the Food and Drug Administration (FDA) for the treatment of stifle joint diseases [163].

## 3. Conclusions

The management of stifle joint disorders in companion animals remains a significant challenge, as no current therapeutic approach can fully solve these conditions. Regenerative medicine offers a promising avenue for improving treatment outcomes, with a range of emerging therapies showing potential to alleviate symptoms and promote joint health. Among these, hyaluronic acid (HA) and platelet-rich plasma (PRP) have been widely used as intra-articular treatments to enhance joint lubrication, reduce inflammation, and provide symptomatic relief. Although effective for some cases, these treatments primarily address the symptoms rather than the underlying pathology of joint degeneration. Interleukin-1 receptor antagonist protein (IRAP), autologous conditioned serum (ACS), and autologous protein solution (APS) represent the next generation of regenerative therapies. These non-platelet hemoderivatives target inflammatory pathways, offering more disease-modifying effects by inhibiting key mediators of joint inflammation. More recently, the MSC-derived secretome has emerged as an innovative, cell-free approach that leverages the diverse bioactive factors secreted by MSCs to support tissue repair and modulate inflammation. Despite their potential, these therapies—HA, PRP, IRAP, ACS, APS, and the secretome—still require further clinical validation to develop standardized protocols, optimize dosing regimens, and confirm their long-term safety and efficacy.

## Figures and Tables

**Table 4:** Characteristic features of clinical and pre-clinical studies evaluating the therapeutic efficacy and safety of stifle intra-articular injection of secretome-based therapies for the treatment of joint diseases in companion animals.

Disease Model	Animals	Mode of Application	Findings	Ref
Spontaneous OA	Dog	Group 1 (*n* = 3) → 2 intra-articular injections of lyosecretome (in the right joint) at 40-day intervals Control group (*n* = 3) → 2 intra-articular injections placebo (in the left joint) at 40-day intervals	Lysosecretome did not induce systemic adverse reactions in terms of lameness and pain worsening.	[161]

OA—osteoarthritis.

## Data Availability

No new data were created or analyzed in this study.
